# The efficacy of peripheral nerve block on postoperative catheter-related bladder discomfort in males: A systematic review and meta-analysis

**DOI:** 10.3389/fsurg.2023.1099628

**Published:** 2023-02-03

**Authors:** Xingjun Bao, Ming Liu, Jie Li, Huibao Yao, Hongquan Liu, Gonglin Tang, Xiaofeng Wang, Zhongbao Zhou, Jitao Wu, Yuanshan Cui

**Affiliations:** ^1^ Second Clinical Medical College, Binzhou Medical University, Yantai, China; ^2^ Department of Urology, The Affiliated Yantai Yuhuangding Hospital of Qingdao University, Yantai, China; ^3^ Department of Anesthesiology, The Affiliated Yantai Yuhuangding Hospital of Qingdao University, Yantai, China; ^4^ Department of Urology, Beijing TianTan Hospital, Capital Medical University, Beijing, China

**Keywords:** peripheral nerve block, CRBD, VAS, PONV, incidence, meta-analysis

## Abstract

**Objective:**

To determine the efficacy of peripheral nerve block (PNB) in preventing postoperative catheter-related bladder discomfort (CRBD).

**Methods:**

Up to July 1, 2022, the PubMed, Embase and Cochrane Central Register of Controlled Trials databases were searched, and all articles that met the PICOS (Patient, Intervention, Comparator, Outcome, Study design) criteria were enrolled. The included trials were evaluated using the Cochrane Collaboration's tool. Patients in the block group received bilateral PNB, while those in the non-block group did not need any additional procedure or simply achieved “sham block”. CRBD was quantified using the visual analog scale (VAS) score, which was questioned and recorded at 0–1 h, 1–2 h, 4–8 h, 8–12 h and 12–24 h intervals. The incidences of CRBD, moderate to severe CRBD and postoperative nausea and vomiting (PONV) were meta-analysed.

**Results:**

Six trials with a total of 544 patients were considered. First, the block group had a lower incidence of CRBD than the non-block group at 0–1 h (OR 0.22; 95% CI, 0.18–0.08; *P* < 0.0001), 1–2 h (OR 0.14; 95% CI, 0.08–0.26; *P* < 0.00001), 4–8 h (OR 0.27; 95% CI, 0.13 to 0.58; *P* < 0.0008) and 8–12 h (OR 0.51; 95% CI, 0.30 to 0.87; *P* = 0.01). Second, the block group showed a lower incidence of moderate to severe CRBD than the non-block group at 0–1 h, 1–2 h and 4–8 h, and the ORs were 0.12 (95% CI, 0.03 to 0.49; *P* = 0.003), 0.17 (95% CI, 0.08 to 0.37; *P* < 0.00001) and 0.29 (95% CI, 0.15 to 0.55; *P* = 0.0002),respectively. Finally, the block group was significantly associated with a decreased incidence of PONV (OR, 0.14; 95% CI, 0.05 to 0.39; *P* = 0.0002).

**Conclusion:**

This meta-analysis suggested that PNB markedly reduced the incidence and severity of early postoperative CRBD and decreased the occurrence of PONV.

## 1. Introduction

Various surgical procedures, particularly urology, mostly require an indwelling urethral catheter postoperatively. The importance of indwelling urethral catheters is self-evident, yet most patients frequently experience varied degrees of catheter-related bladder discomfort (CRBD), a phenomenon that appears to be more prevalent in male patients (1–3). CRBD can intensify postoperative pain and prolong the hospital stay of patients, impeding the progress of enhanced recovery after surgery (ERAS), and there are even reports in the literature that CRBD can increase the incidence of postoperative complications, including surgical incision dehiscence and hemorrhage (4, 5).

Not surprisingly, the incidence of CRBD can be as high as 90% in patients after transurethral resection of bladder tumors (6). The clinical manifestation of CRBD is a burning sensation that spreads from the suprapubic region to the penis, and some patients even develop an urge to pull out the urinary catheter. Moreover, some patients also present with symptoms similar to overactive bladder (OAB) (7, 8). Given that CRBD is primarily caused by involuntary contraction of bladder smooth muscle mediated by muscarinic receptors, a variety of antimuscarinic medications have developed. Furthermore, some antiepileptic agents, selective *α*_2_-adrenoceptor agonists and nonsteroidal anti-inflammatory drugs (NSAIDs) have been demonstrated to be effective (4, 5). In recent years, research on peripheral nerve block (PNB) for the prevention of CRBD has become a hotspot to decrease pharmacological side effects.

A prospective study showed that PNB can effectively relieve CRBD and minimize analgesic requirements, whereas Weinberg and his colleagues were skeptical (9, 10). Therefore, for the first time, we conducted a meta-analysis to assess the efficacy of PNB for the prevention of postoperative CRBD.

## 2. Methods

This systematic review and meta-analysis followed the latest Preferred Reporting Items for Systematic Reviews of Interventions (PRISMA 2020) statements and the completed PRISMA 2020 checklist is presented in the supplementary file.

### 2.1. Search strategy

**Figure 1:**
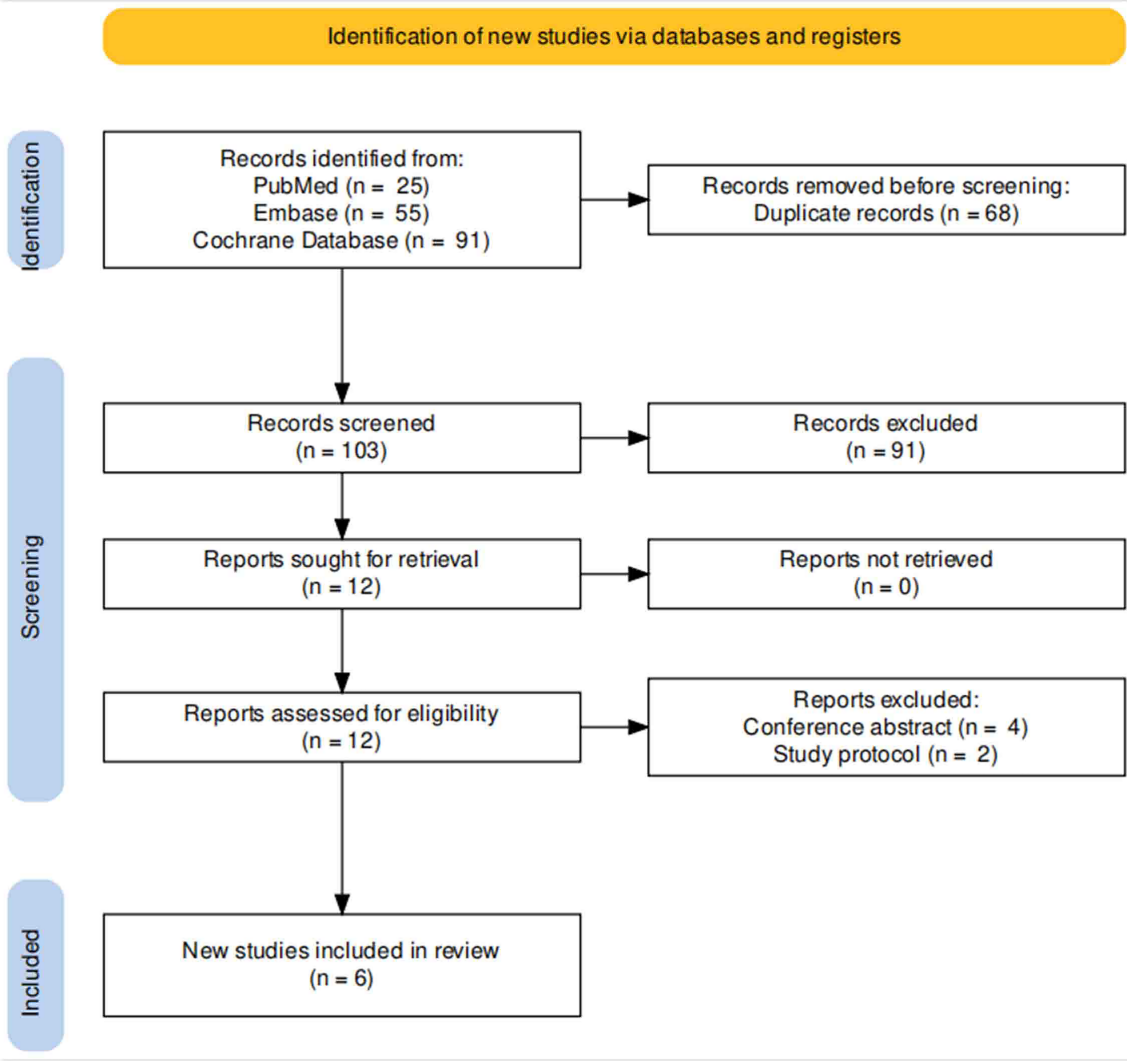
Flowchart of the literature search.

Up to July 1, 2022, search phrases (“catheter discomfort” and” block “) were retrieved from the PubMed, Embase and Cochrane Central Register of Controlled Trials databases, and all literature was systematically reviewed. Two authors utilized PICOS (Patient, Intervention, Comparator, Outcome, Study design) criteria to include relevant randomized controlled trials (RCTs). Case reports, editorials, conference abstracts, study protocols, and non-English records were not considered. In addition, we gathered articles of interest from the included articles’ reference lists. If the two reviewers disagreed, they consulted with a third author to reach a consensus. Figure 1 depicts the PRISMA flow diagram.

### 2.2. Inclusion criteria

The detailed PICOS criteria are presented in Table 1. Furthermore, all enrolled patients randomly performed the experimental or control interventions after surgery. After arrival at the post-anesthesia care unit (PACU), the visual analog scale (VAS) score was inquired and recorded at 0–1 h, 1–2 h, 4–8 h, 8–12 h and 12–24 h, respectively. The severity of CRBD was classified as none (VAS 0), mild (VAS 1–3), moderate (VAS 4–7) and severe (VAS 8–10). Patients were randomly allocated to one of two groups: block or non-block, with the former producing bilateral PNB *via* local anesthesia and the latter having no any additional procedure or simply replacing local anesthetics with saline to achieve “sham block”.

**Table 1:** Search strategy according to population, intervention, comparison, outcome, and study design.

	Population	Intervention	Compariosn	Outcomes	Study design
Inclusion Criteria	Adult male patients; ASA grading (I–III); Indwelling catheter after surgery; Without respiration, circulation disorders, or chronic pain;	Pudendal nerve block; Dorsal penile nerve block; Caudal block; TAPB;	Shame block using an injection of saline; Without any treatment;	The incidence of CRBD and moderate to severe CRBD; The incidence of PONV; The incidence of need for analgesic and opioid consumption in 24 h after surgery;	Randomized controlled trials
Exclusion Criteria	Age lower than 18; Any contraindication to the anaesthetic protocol; Bladder or rectal dysfunction, severe motor neuropathy, mental disorders, or bleeding tendency; History of indwelling- catheterization and pre-existing infection at the site of injection;	Not performed	Not performed	The VAS score of pain related to catheter; Changes in heart rate and mean blood pressure; The intestines function recovery time;	Letters, comments, reviews, conference abstract, study protocol and qualitative studies

ASA, American society of anesthesiologists; TAPB, transversus abdominis plane block; VAS, visual analog scale; CRBD, catheter-related bladder discomfort; PONV, post-operative nausea and vomiting.

### 2.3. Quality assessment

The Cochrane Collaboration's tool, which comprised seven aspects: random sequence generation, allocation concealment, blinding of participants and personnel, blinding of outcome assessment, incomplete outcome data, selective reporting and other potential bias, was used to evaluate the included RCTs (11). The above assessments were accomplished separately by two authors, and disagreements among reviewers were settled by consensus.

### 2.4. Data extraction

The following data were obtained from the included studies: (1) First author's name and year of publication; (2) nation of study and sample size in each RCT; (3) nerve block modalities and drugs; (4) anesthesiology and types of surgery; (5) operative duration; (6) catheter size and inflated volume; (7) the incidence of CRBD and moderate to severe CRBD; (8) the incidence of postoperative nausea and vomiting (PONV); and (9) the incidence of need for analgesic and opioid consumption 24 h after surgery.

### 2.5. Statistical analysis and meta-analysis

The interquartile range was converted into the mean and standard deviation (SD) using the quantile estimation (QE) methodology recently developed by McGrath and colleagues (12). Continuous variables were reported as the mean difference (MD) with 95% confidence interval (CI), whilst dichotomous variables were described using odds ratio (OR) and CI. Heterogeneity among the included studies was determined by the I-square (*I*^2^) and *Q* tests. A random-effect model was employed if the heterogeneity was significant (*P* < 0.05 and *I*^2 ^≥ 50%); otherwise, a fixed-effect model was used. A *P* value of less than 0.05 was judged statistically significant for the summary effect. Quality assessment and outcome analysis of the included RCTs were performed with RevMan v.5.4.0 (Cochrane Collaboration, Oxford, UK).

## 3. Results

### 3.1. Characteristics of the individual studies

A total of 171 articles were retrieved. After screening titles and abstracts, 159 papers were rejected. After full-text access, four conference abstracts and two study protocols (13, 14) were removed, leaving six studies for inclusion in the meta-analysis. Three RCTs used dorsal penile nerve block (9, 10, 15), one caudal block (16), one pudendal nerve block (17), and one transvs. abdominis plane block (18) as nerve block methods. The patients with indwelling urinary catheters underwent various surgical interventions, the majority of which were prostatic operations, a few of which were bladder procedures, and a few of which were nonurologic surgeries. Table 2 summarizes the detailed features of the included studies.

**Table 2:** Baseline characteristics of the included studies.

Study	Country	Gender	Study groups		Sample	location method of nerve block	Types of surgery	Catheter size	Inflated balloon	Inflated volume	Anesthesiology	Duration operation (M ± SD, minutes)
			Block group	Non-block group								
Göger et al. (2020)	Turkey	Men	BDPNB: 10 ml of 0.25% bupivacaine	No additional procedure	40/40	Ultrasound-guided	TURP	20 Fr	Gas	40 ml	Spinal	50 ± 24 vs. 51 ± 18
Li et al. (2017)	China	Men	BPNB: 10 ml of 0.5% ropivacaine	No additional procedure	86/89	Anatomic landmarks	TURP, TURBT	20 or 22 Fr	Water	30 ml	General	55 ± 36 vs. 48 ± 32
Maquoi et al. (2016)	Belgium	Men	TAPB: 20 ml of 0.38% levobupicavaine	TAPB: 20 ml of 0.375% saline	34**/**33	Ultrasound-guided	Open prostate surgery	Not mentioned	Not mentioned	Not mentioned	General	Not mentioned
Li et al. (2016)	China	Men	BDPNB: 15 ml of 0.33% ropivacaine	No additional procedure	29/29	Anatomic landmarks	Elective liver surgery or limb surgery	18 Fr	Saline	10 ml	General	134 ± 55 vs. 124 ± 56
Weinberg et al. (2014)	USA	Men	BDPNB: 20 ml of 0.25% bupivacaine	DPNB: 20 ml of saline	56/60	Anatomic landmarks	RARP with bilateral pelvic lymph node dissection	18 Fr	Not mentioned	Not mentioned	General	166 ± 80 vs. 186 ± 79
Tsuchiya et al. (2013)	Japan	Men	SSCB: a mixture of 0.3% ropivacaine (8 ml) and fentanyl (100 ug)	No additional procedure	24/24	Ultrasound-guided	Cervical laminoplasty	16 Fr	Water	10 ml	General	146 ± 43 vs. 139 ± 37

Abbreviations: BDPNB, bilateral dorsal penile nerve block; BPNB, bilateral pudendal nerve block; TAPB, transversus abdominis plane block; SSCB, single shot caudal block; TURP, trans-urethral resection of prostate; TURBT, trans-urethral resection of bladder tumor; RARP, robotic-assisted radical prostatectomy; M ± SD, mean ± standard deviation.

### 3.2. Quality assessment of the individual studies

**Figure 2:**
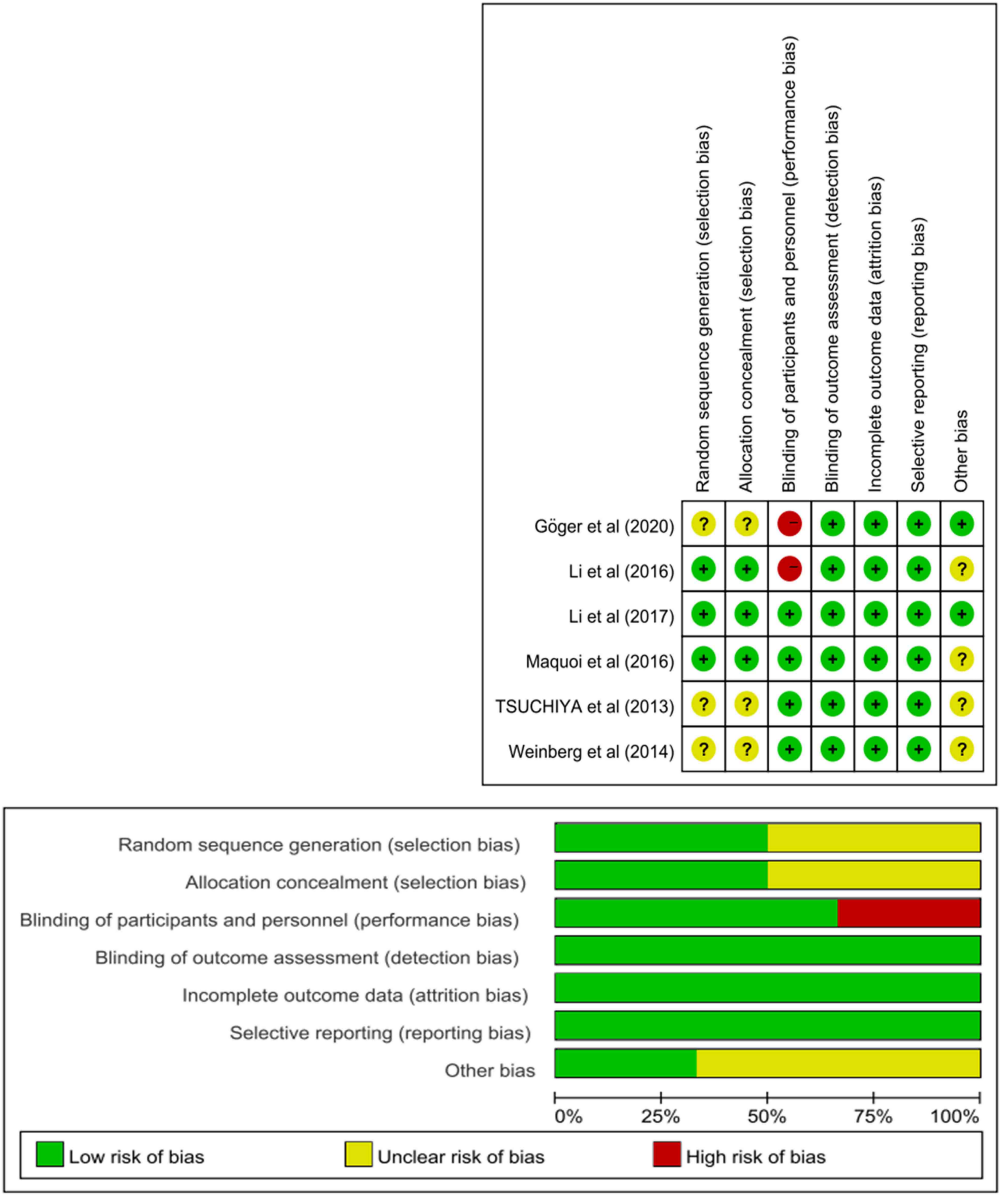
Risk of bias summary and graph of the included studies.

Figure 2 shows the risk-of-bias summary and graph. Although all of the included studies were RCTs, only three of them (15, 17, 18) detailed their randomization protocols. Two RCTs (9, 15) did not use the double-blind method, and performance bias was rated as a high risk of bias. The others were classified as having a low or unclear risk of bias.

### 3.3. The efficacy of PNB

VAS scores of CRBD at 0–1 h (arrival in the PACU), 1–2 h, 4–8 h, 8–12 h and 12–24 h in the block and non-block groups were inquired and recorded, and a meta-analysis was performed to investigate the incidences of CRDB and moderate to severe CRBD between the two groups and to evaluate the effectiveness of PNB in preventing CRDB.

#### 3.3.1. The incidence of CRBD

**Figure 3:**
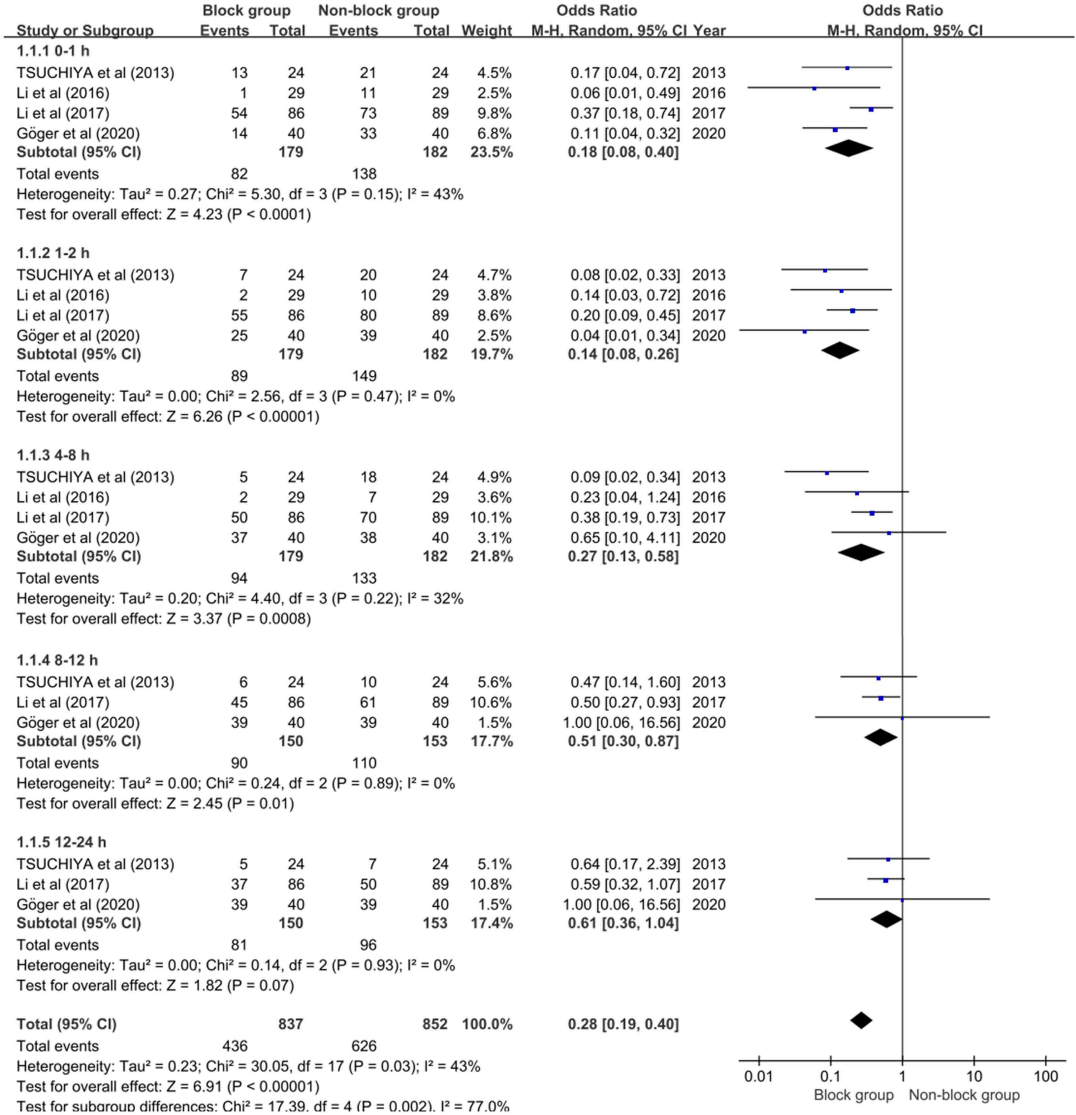
Forest plot illustrating the incidence of CRBD.

Four studies integrating 361 individuals (179 in the block group and 182 in the non-block group) were enrolled in the meta-analysis to assess the influence of PNB on the incidence of CRBD. Because of the considerable heterogeneity (*P* = 0.03, *I*^2 ^= 43%) among studies, the random-effect model was used. Individuals who received a pudendal nerve block had a lower incidence of CRBD than those who did not receive a pudendal nerve block at 0–1 h (OR 0.22; 95% CI, 0.18–0.08; *P* < 0.0001), 1–2 h (OR 0.14; 95% CI, 0.08–0.26; *P* < 0.00001), 4–8 h (OR 0.27; 95% CI, 0.13 to 0.58; *P* < 0.0008) and 8–12 h (OR 0.51; 95% CI, 0.30 to 0.87; *P* = 0.01), while there was no significant difference between the two groups in the incidence of CRBD at 12–24 h (OR 0.61; 95% CI, 0.36 to 1.04; *P* = 0.07) (see Figure 3). According to the findings of this meta-analysis, PNB can dramatically lower the incidence of postoperative CRBD in male patients.

#### 3.3.2. The incidence of moderate to severe CRBD

**Figure 4:**
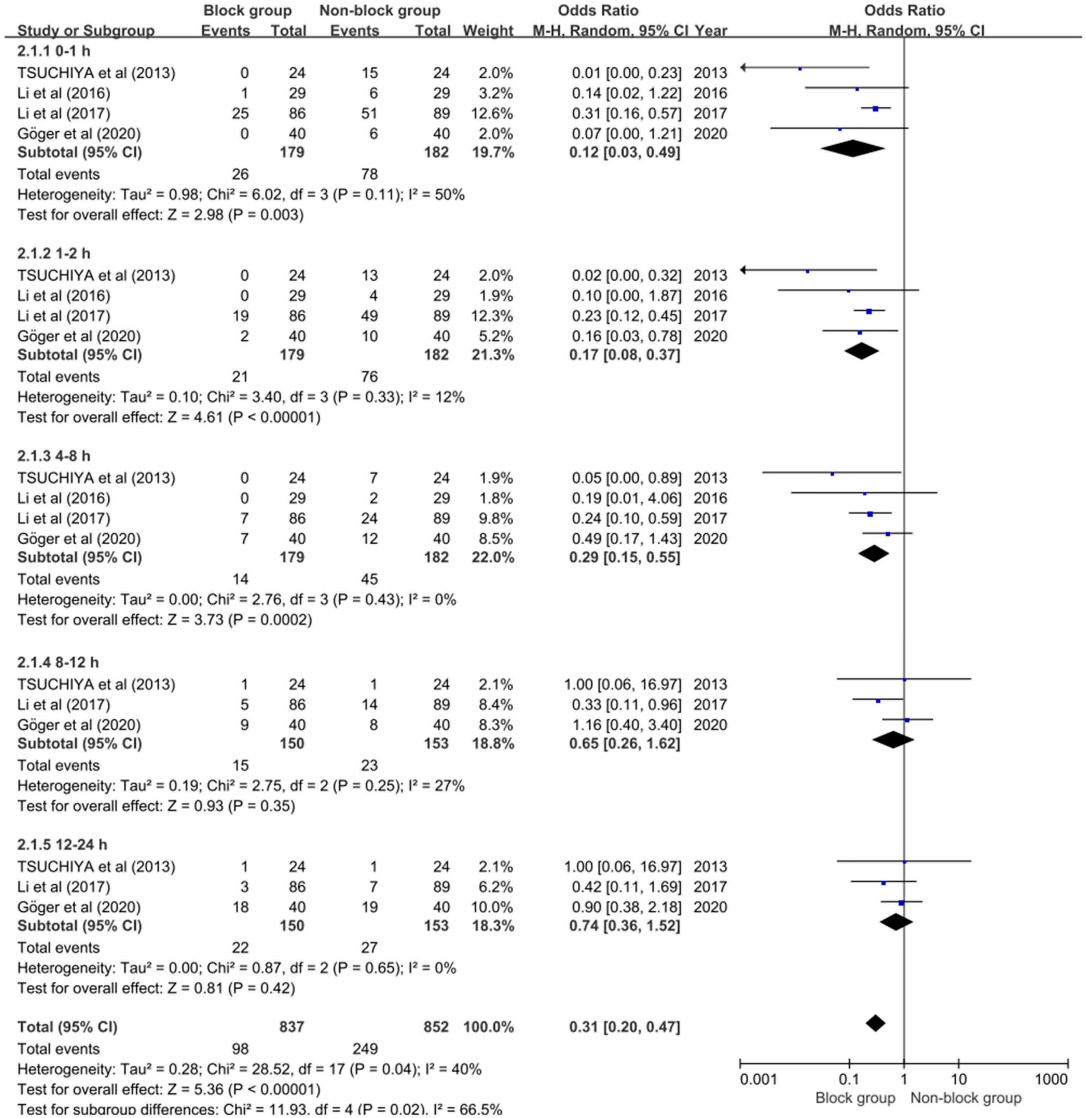
Forest plot illustrating the incidence of moderate to severe CRBD.

Four studies integrating 361 individuals (179 in the block group and 182 in the non-block group) were enrolled in the meta-analysis to further evaluate the impact of PNB on the incidence of moderate to severe CRBD. There was considerable heterogeneity (*P* = 0.04, *I*^2 ^= 40%) among studies, hence the random-effect model was used. The block group showed a lower incidence of moderate to severe CRBD than non-block group at 0–1 h, 1–2 h and 4–8 h, and the ORs were 0.12 (95% CI, 0.03 to 0.49; *P* = 0.003), 0.17 (95% CI, 0.08 to 0.37; *P* < 0.00001) and 0.29 (95% CI, 0.15 to 0.55; *P* = 0.0002) respectively. Conversely, there was no significant difference at 8–12 h (OR 0.65; 95% CI, 0.26 to 1.62; *P* = 0.35) and 12–24 h postoperatively (OR 0.74; 95% CI, 0.36 to 1.52; *P* = 0.42), as shown in Figure 4. These findings showed that PNB significantly reduced the incidence of postoperative moderate to severe CRBD in male patients.

#### 3.3.3. The incidence of PONV

Three studies incorporating 205 patients (103 in the block group and 102 in the non-block group) revealed adifference in the occurrence of PONV. There was no heterogeneity (*P* = 0.56, *I*^2 ^= 0%) among studies, so the fixed-effect model was used for the meta-analysis. As shown in Figure 4, the non-block group was significantly associated with a higher incidence of PONV (OR, 0.14; 95% CI, 0.05 to 0.39; *P* = 0.0002). We concluded that PNB might greatly lower the incidence of PONV in male patients.

#### 3.3.4. The opioid need in 24 h after surgery

First, two RCTs involving 138 patients reported the incidence of need for analgesics in 24 h after surgery. Our pooled analysis indicated that although there was a difference between the two groups in the incidence of need for analgesics, there was no statistical significance (OR 0.70; 95% CI, 0.33 to 1.47; *P* = 0.34).In addition, two RCTs involving 183 patients reported opioid consumption in 24 h after surgery. No significant difference was shown between the two sets (OR −6.33; 95% CI, −17.05 to 4.38; *P* = 0.25).

## 4. Discussion

CRBD, as an unavoidable clinical entity, not only afflicts patients but also frequently makes clinicians lose what to do. Studies on CRBD has never ended, and scholars have been striving for a better solution to handle this perplexing problem. Antimuscarinic agents are the first-line drugs for treating CRBD since it has long been recognized that CRBD is induced by involuntary contraction of bladder smooth muscle and that the neurotransmitter released in this physiological process isacetylcholine. Solifenacin, darifenacin, butylscopolamine, oxybutynin and tolterodine are antimuscarinic medicines with demonstrated effectiveness (4, 5). Nevertheless, some patients had adverse effects such as dry mouth, constipation, sleepiness and blurring of vision, resulting in poorer patient compliance (19). Emerging antiepileptic drugs such as gabapentin and pregabalin, as well as anesthetics like ketamine, have been demonstrated to significantly relieve CRBD, albeit with sedation (5, 20). Moreover, a recent meta-analysis revealed that subhypnotic doses of ketamine can decrease the incidence and severity of postoperative CRBD without causing significant adverse effects (21). Furthermore, paracetamol, as an inhibitor of prostaglandin synthesis, had some effect on CRBD, although there were fewer reports (22).

Nerve block technology has evolved with the advent of ultrasound devices. Physiologically, impulses in afferent nerves derived in the urethra and bladder triangle travel from the sacral nerves (S2–4) to the sacral segments of the spinal cord and eventually to the pudendal nerves and their branches, triggering urethral muscles and sphincters of the perineum and pelvic floor to contract (23). The mechanism of CRBD is strongly intertwined with this reflex arc, and the presence of a urinary catheter induces mechanical stimulation of the bladder or urethra, which in turn drives involuntary spasms of bladder smooth muscle (8). Based on this logic, blocking either region on this reflex arc would help avoid CRBD.

Pudendal nerve block and dorsal penile nerve block are now the most well-known methods of PNB, with the latter being the terminal branch of the pudendal nerve. In fact, pudendal nerve or branch block was initially employed to treat anal and urethral sphincter incompetence (24). Additionally, pudendal nerve block can also be used to relieve pain after vaginal birth, vaginal repair, circumcision and hypospadias (17). Weinberg and colleagues initially conducted a double-blind trial in 2014 to assess the effect of dorsal penile nerve block on CRBD following robot-assisted radical prostatectomy. Unfortunately, the evidence gathered did not support the efficacy of dorsal penile nerve block (10). In contrast, a prospective, randomized, controlled experiment indicated that dorsal penile nerve block was both safe and effective in preventing postoperative CRBD (15). Herein, we performed this meta-analysis with a total of six articles, and our study proved that PNB significantly decreased the incidence of CRBD, moderate to severe CRBD and PONV in patients with urinary catheters, especially within 8 h after surgery.

It is noteworthy that caudal block is the first to be utilized to treat CRBD. In 2013, Tsuchiya et al. discovered that ultrasound-guided caudal block might safely diminish CRBD in male patients following cervical laminoplasty (16). However, the caudal block procedure has been so dangerous, especially in obese or older patients, that clinicians have been overwhelmed. Caudal block can result in repeated punctures, hematomas, neurological damage, and infections due to anatomical variances of the sacral hiatus, even with ultrasound guidance (16, 25). In addition, a randomized controlled trial sought to validate the effectiveness of transvs. abdominis plane block in alleviating CRBD and pain in patients following open prostatectomy, however the findings were discouraging (18). Similarly, the current meta-analysis failed to demonstrate that nerve blocks reduced postoperative analgesic consumption in patients, but this cannot be ignored due to the small number of studies included in this meta-analysis. The difference in the incidence of CRBD and moderate to severe CRBD between the block group and non-block group vanished within 8 h postoperatively, possibly due to the anesthetic effect of the PNB wearing off. As a result, the timing of nerve blocks should be dictated by the length of the surgery (16). Currently, most studies have revealed no major complications with the use of PNB, which may benefit from ultrasound technology. Some self-limiting complications, including urine retention, mild bleeding, fever, tachycardia, or hypotension, usually heal on their own. Notably, hematoma, pudendal nerve injury, vascular injury, rectal perforation, muscular weakness or numbness in the region of nerve block, and urine or fecal incontinence are all unusual complications of PNB (26, 27). Once those issues occur, pharmacological or even surgical intervention may be warranted. A previous study reported that the risks of complicated fever, urinary retention, and hemorrhage in the PNB group were 3.4%, 1.7%, and 1.7%, respectively (28). Although the effectiveness of PNB in reducing CRBD is widely recognized, the risks of nerve block should not be neglected.

Despite the fact that all RCTs were included in this study, it had several flaws. First, the anesthetics used in the nerve block were not uniform, and whether this had an influence on our results had to be determined. Although the VAS score is frequently used to assess patients' symptoms, it is difficult to discern between CRBD and catheter-related pain or incision-related pain, so appropriate questionnaires to assess CRBD are desperately needed (5). It is also an important confounding element that we do not have access to whether antimuscarinic medications were used in included patients. Second, a prospective observational study (29) indicated that the type of surgery was a predictor of CRBD severity, and prostate surgery seemed to cause more severe CRBD than bladder or non urological surgery. However, the factor differed in our meta-analysis, which might be a significant confounding factor. Finally, the inconsistency of catheter diameter and balloon inflation are bound to impact our findings, but the subjects in this study were all male patients, which eliminated gender differences and made our findings more convincing, as a prospective observational study found that male sex and catheter diameter greater than or equal to 18 Fr were significant predictors of CRBD (1). To the best of our knowledge, this is the first systematic review and meta-analysis of the effectiveness of PNB in the treatment of CRBD.

## 5. Conclusion

In conclusion, our meta-analysis demonstrated that PNB is an effective measure for the prevention of postoperative CRBD in male patients, as it reduces the incidence of CRBD, moderate to severe CRBD and PONV, particularly within 8 h after surgery. Nevertheless, a pooled analysis with a larger sample to validate our conclusions is warranted.

## Data Availability

The original contributions presented in the study are included in the article/Supplementary Material, further inquiries can be directed to the corresponding author/s.
